# A cryptic species of *Onchocerca* (Nematoda: Onchocercidae) in blackflies (*Simulium* spp.) from southern California, USA

**DOI:** 10.1186/s13071-018-3133-9

**Published:** 2018-10-16

**Authors:** Guilherme G Verocai, Kimberly J Nelson, R Trey Callahan, Joseph Wakoli Wekesa, Hassan K Hassan, Eric P Hoberg

**Affiliations:** 1 Department of Infectious Diseases, College of Veterinary Medicine, University of Georgia, 501 D.W. Brooks Drive, Athens, GA 30602 USA; 2 Department of Global Health, College of Public Health, University of South Florida, 3720 Spectrum Boulevard, Tampa, FL 33612 USA; 3 San Gabriel Valley Mosquito and Vector Control District, 1145 N. Azusa Canyon Rd, West Covina, CA 91790 USA; 4 Coachella Valley Mosquito and Vector Control District, 43420 Trader Place, Indio, CA 92201 USA; 5 School of Veterinary Medicine, Department of Pathobiological Sciences, University of Wisconsin, Madison, WI 53706 USA

**Keywords:** Cervidae, Filarial parasites, Filarioidea, Onchocerciasis, Parasite biodiversity, Vector-borne diseases

## Abstract

**Background:**

Entomological surveillance for pathogens based on molecular screening of putative arthropod vectors such as blackflies (Diptera: Simuliidae) is becoming increasingly important. Surveillance provides a means to understand host and geographical patterns of underestimated biodiversity among North American species of *Onchocerca* and a pathway to identify and track expanding emergence of the zoonotic *Onchocerca lupi*. Herein, we have screened two blackfly species, *Simulium tescorum* and *Simulium vittatum* (*s.l*.), from Los Angeles County, southern California, USA for DNA of filarioid nematodes to better understand species richness and limits within the genus *Onchocerca*.

**Methods:**

A total of 1056 and 378 female blackflies was collected using CO_2_-baited mosquito traps from March to November of 2015 and 2016, respectively. All blackflies during 2015 were individually processed for DNA extraction and PCR targeting of the cytochrome *c* oxidase subunit 1 (*cox*1) of the mitochondrial DNA (mtDNA). Specimens of *S. tescorum* collected in 2016 were processed individually with heads and bodies extracted separately, whereas those of *S. vittatum* (*s.l*.) were processed in pooled samples with heads and bodies extracted separately. A subset of filarioid-positive samples from 2015 and all samples from 2016 were screened using a PCR targeting the NADH dehydrogenase subunit 5 (*nad*5) gene (mtDNA).

**Results:**

In 2015, 356 *S. tescorum* (33.7%) and 683 *S. vittatum* (*s.l*.) (64.7%) were collected, and an additional 17 specimens were not assessed morphologically. In 2016, a total of 378 blackflies was collected. Of these, 43 (11.6%) were *S. tescorum* and 327 (88.4%) were *S. vittatum* (*s.l*.), and an additional 8 specimens were not assessed morphologically. In 2015, *Onchocerca* sequences were detected in 4.8% (*n* = 17) of *S. tescorum* samples, and only one *S. vittatum* (0.15%). In 2016, only a single *S. vittatum* pool was positive for the same cryptic *Onchocerca* species. In phylogenetic comparisons based on *nad*5, the *Onchocerca* sequences from California formed a clade with those isolates in white-tailed deer from upstate New York, suggesting these belong to a single widespread cryptic species.

**Conclusions:**

An uncharacterized species of *Onchocerca* associated with cervid hosts was found in blackflies from southern California. Sequence data demonstrated it is likely conspecific with an unnamed species of *Onchocerca* previously found in white-tailed deer from upstate New York. Current data support recognition of a broad geographical distribution across North America for an apparently cryptic species of *Onchocerca* that is discrete from *O. cervipedis*, considered to be a typical filarioid among cervids. Our data suggest that this cryptic species of *Onchocerca* may infect subspecies of white-tailed deer (*Odocoileus virginianus*), and mule and black-tailed deer (*Odocoileus hemionus*) at temporal latitudes. The blackflies *Simulium tescorum* and *S. vittatum* (*s.l*.) (presumably, *S. tribulatum*) are putative vectors. Discovery of a cryptic complex indicates that species diversity and putative associations for definitive hosts and vectors of *Onchocerca* species in North America must be reassessed.

## Background

*Onchocerca* (Nematoda:;Filarioidea: Onchocercidae) is a genus of parasitic nematodes infecting wild and domestic ungulates, carnivores, and humans globally [1–3]. Despite the medical and veterinary importance of some species, including *Onchocerca volvulus* (Leuckart, 1893), the causative agent of human onchocerciasis or river blindness, and the emerging zoonotic *Onchocerca lupi* Rodonaja 1967, the causative agent of canine ocular onchocercosis, most species associated with domestic and wild ungulate hosts have not been directly linked to disease [1]. Species of *Onchocerca* are transmitted to mammalian definitive hosts by dipteran arthropod biological vectors, including species of blackflies (Simuliidae) and biting midges (Ceratopogonidae) [2].

*Onchocerca* is a speciose genus encompassing 34 valid species [1]. There is evidence, however, for underestimated species richness and the potential for unrecognized cryptic diversity [4–7]. For instance, three species have been described from Japan in the last four decades, i.e. *Onchocerca suzukii* Yagi, Bain & Shoho, 1994 in the Japanese serow *Capricornus crispus* (Temminck) [6], *Onchocerca eberhardi* Uni & Bain, 2007 in the sika deer *Cervus nippon* Temminck [4], and *Onchocerca takaokai* Uni, Fukuda & Bain, 2015 in the Japanese wild boar *Sus scrofa leucomystax* Temminck [5].

In addition to these observations from Asia, there is molecular evidence for a cryptic species infecting the white-tailed deer *Odocoileus virginianus* (Zimmermann) from North America [7]. Until recently, *Onchocerca cervipedis* Wehr & Dikmans, 1935 was the only recognized species infecting wild ungulates across North and Central America, and it remains the only formally described species [8–11]. Its reported host range includes five cervids: the white-tailed deer; the mule deer *Odocoileus hemionus* (Rafinesque); moose *Alces americanus* (Clinton); elk or wapiti *Cervus canadensis* Erxleben; and caribou *Rangifer tarandus* (L.); and also, the antilocaprid pronghorn *Antilocapra americana* Ord [8, 10–19]. Consistent with discovery of cryptic diversity among other ungulate nematodes, *O. cervipedis* may constitute a complex of species, as it represents a geographically widespread parasite with a considerable host range. Potential species limits and diversity for *Onchocerca* in conjunction with host range, and geographical associations must be revisited. Transmission pathways, vector diversity and associations also require reconsideration.

Recent evidence for underestimated diversity among North American species of *Onchocerca*, in conjunction with the emergence of the zoonotic *O. lupi*, have increased interest in entomological surveillance for these parasites. Molecular-based screening of putative arthropod biological vectors, including blackflies (Diptera: Simuliidae) for DNA of filarial nematodes is a powerful methodology to reveal occurrence of cryptic diversity [20]. Herein, we have screened two blackfly species, *Simulium tescorum* Stone & Boreham and *Simulium vittatum* (*s.l*.), from Los Angeles County, southern California, USA, for DNA of filarial nematodes. We provide some initial observations about diversity and geographical distribution of a poorly understood assemblage of species within *Onchocerca* from North America. Also, we discuss the role of these blackflies as vectors, the putative association with mule deer as definitive hosts, and provide insights about the historical biogeography of a cryptic *Onchocerca* species originally documented in white-tailed deer from New York [7].

## Methods

### Blackfly collection

Los Angeles County (LAC), California, is the most populous county in the USA with a population of over 10 million inhabitants in an area of 12,310 km^2^, with a density of 800 people/m^2^. Elevation varies from sea level to 3069 m encompassing heterogeneous habitats supported by a Mediterranean climate with usually hot and dry summers. Surveillance was targeted in this region based on the endemic occurrence of the zoonotic *O. lupi* [20], and the potential occurrence of other species of *Onchocerca* that may be associated with mule deer (*Odocoileus hemionus*), the only endemic cervid in the region [21].

Adult female blackflies were collected through collaborations with the San Gabriel Valley Mosquito and Vector Control District (SGVMVCD) and the Greater Los Angeles County Vector Control District (GLACVCD) using mosquito day traps baited with CO_2_. Flies were collected from various sites by SGVMVCD, and GLACVCD personnel from March to November of 2015 and 2016.

### Morphological identification of blackflies

Blackflies collected by the SGVMVCD were morphologically identified to species/species-complex level according to taxonomic keys [22], and stored frozen at -80 °C until analysis. Specimens collected by GLACVCD were not identified to species and stored in 70% ethanol. Voucher specimens of blackflies were not retained or archived in a museum repository due to the destructive nature of sampling that was required for molecular screening in the present study.

### Molecular screening for *Onchocerca* spp. DNA

#### 2015 collections

All blackflies collected in 2015 were processed individually for DNA extraction using the Qiagen DNeasy® Blood & Tissue kit (Qiagen, Valencia, CA, USA). Briefly, all flies were macerated with sterile plastic pestles within an Eppendorf tube, and homogenized with ATL buffer, and proteinase K. Samples were then incubated in a dry heat block for 45 min at 56 °C, and then centrifuged for 5 min. at 8000× *g*. The supernatant of each sample was transferred into new tubes. Extractions were processed in the automated, low throughput robotic workstation (QIAcube, Qiagen, Valencia, CA, USA) using the manufacturer’s protocol for tissue.

Polymerase chain reaction was performed on the blackfly DNA extracts in search of the presence *Onchocerca* sp. DNA. Multiple genetic markers target the mitochondrial genes of filarial worms in search of *Onchocerca* sp. These include the NADH dehydrogenase subunit 5 (*nad*5) gene and the cytochrome *c* oxidase subunit 1 (*cox*1) gene. The PCR protocols used for these genetic markers are listed below; GOTAQ™ DNA polymerase was used in these reactions. Primers targeting the *cox*1 were: HFCOI-F (5'-TGT TGC CTT TGA TGT TGG GG-3') and HFICOI-R (5'-GGA TGA CCG AAA AAC CAA AAC AAG-3') [23]. Cycling conditions for the *cox*1 consisted of 95 °C for 2 min, followed by 40 cycles of 95 °C for 30 s, 52 °C for 50 s, and 72 °C for 30 s, and a final extension at 72 °C for 5 min.

It was necessary to amplify and sequence the *nad*5 region of the mitochondrial DNA of a subset of the confirmed *Onchocerca*-positive blackflies from the 2015 collection, because there was no information available on the *cox*1 region of the cryptic *Onchocerca* found in white-tailed deer from upstate New York [7]. Briefly, *nad*5 PCR reactions followed the same protocol, using the primers ND5-Ov5A-F (5'-TTG GTT GCC TAA GGC TAT GG-3') and ND5OvC-R (5'-CCC CTA GTA AAC AAC AAA CCA CA-3') [24]. Cycling conditions for the ND5 region consisted of 95 °C for 2 min, followed by 35 cycles of 95 °C for 30 s, 50 °C for 45 s, and 72 °C for 30 s, and a final extension at 72 °C for 5 min.

#### 2016 collections

All 2016 extractions followed the same protocol of maceration and incubation in ATL buffer and proteinase K; however, all following steps of the DNA extraction protocols were performed manually following the manufacturer’s protocol.

For specimens morphologically identified as *Simulium tescorum*, we performed individual DNA extractions, for each head and body, separately. Because of the high number of *Simulium vittatum*-complex specimens, heads and bodies were extracted separately, and samples consisted from individual flies (i.e. single fly caught in a trap) to pools of up to 10 flies. Pools consisted of flies caught in the same trap, separated by species. All 2016 samples were screened using the above described PCR reaction targeting the *nad*5 region of the mtDNA.

### Blood-meal analysis

A subset of 36 *S. tescorum* from the 2015 collections, considered potentially engorged, were screened for the host blood meal using a PCR targeting the *16S* rRNA of vertebrates [25]. PCR reactions followed the previous mentioned protocol, using the primers L2513-F (5'-GCC TGT TTA CCA AAA ACA TCC-3') and H2714-R (5'-CTC CAT AGG GTC TTC TCG TCT T-3'). Cycling conditions consisted of 95 °C for 4 min, followed by 35 cycles of 95 °C for 30 s, 57 °C for 30 s, and 72 °C for 30 s, and a final extension at 72 °C for 10 min.

### Sequencing

All PCR products of samples that produced amplicons visualized in the agarose gel, were column purified using the Kit Cycle Pure EZNA kit (Omega Bio-Tek, Norcross, GA, USA) following the manufacturer’s protocol. Products were then directly sequenced using their respective forward and reverse primers using BigDye Terminator Cycle sequencing.

### Phylogenetic analysis

All sequences were edited individually, and aligned by Clustal W in MEGA 7 [26]. Phylogenetic trees of the *cox*1 (432 bp) and *nad*5 (427 bp) sequences were constructed using the Maximum Parsimony method, with 2000 bootstrap replicates. We have included in the phylogenetic analyses sequences of *Onchocerca* species available in GenBank. Sequences of *Dirofilaria immitis* (Leidy, 1856) and *Dirofilaria repens* Railliet & Henry, 1911, which also belong to the family Onchocercidae, were included as outgroups [27].

### Taxonomy of simuliid vectors and mammalian hosts for *Onchocerca*

The taxonomy of blackflies followed the most recent and comprehensive literature [22, 28]. Taxonomy of artiodactyl mammalian hosts for *Onchocerca* and other filarioid taxa followed Grubb (2005) [29].

## Results

### Collections

**Fig. 1:**
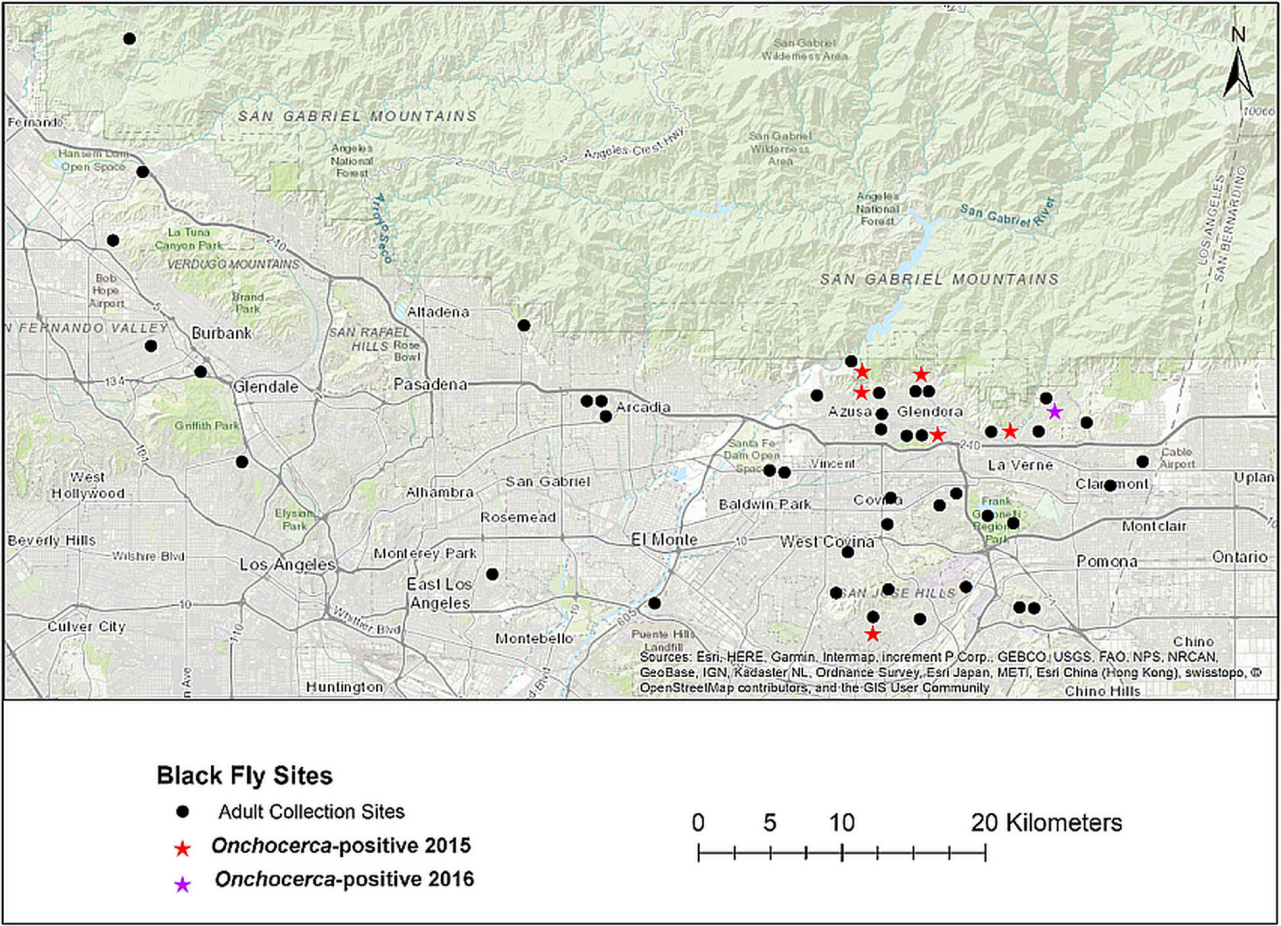
Adult blackfly collection sites in Los Angeles County, California. Adult blackflies that tested positive for the uncharacterized *Onchocerca* sp. in 2015 (*n* = 6) are indicated by a red star, and the single *Onchocerca-*positive sample in 2016 (*n* = 1) is indicated by a purple star.

A total of 1056 blackflies was collected during the 2015 collections; of these, 1039 were collected from 34 sites within the SGVMVCD area, with 683 (65.7%) identified as species of the *S. vittatum* (*s.l.)* and 373 (34.3%) as *S. tescorum* (Fig. 1). Seventeen blackflies collected from six sites by the GLACVCD were not morphologically identified to the species/species-complex level.

In the 2016 collections, a total of 378 host-seeking female blackflies was collected from 23 sites in the SGVMVCD area and 8 sites in the GLACVCD (Fig. 1). Out of the 370 blackfly specimens in the San Gabriel Valley, 327 specimens (88.4%) were identified as *S. vittatum*-complex and 43 (11.6%) as *S. tescorum*.

### *Onchocerca* screening

Regarding the 2015 collections, a total of 18 (1.7%) blackflies collected in 2015 was positive for *Onchocerca* DNA. Of these, 17 were *S. tescorum* (4.8%), and one *S. vittatum*-complex (0.2%). The *cox*1 sequences did not match any species of *Onchocerca* available in GenBank. Hence, a subset of these samples had their *nad*5 region amplified and sequenced.

Out of the *S. tescorum* samples, 11 were collected from a single location along the San Gabriel River in Azusa, were *Onchocerca*-positive in March (*n* = 7), July (*n* = 3) and August (*n* = 1). Five *Onchocerca*-positive *S. tescorum* were collected from three locations in Glendora, in the months of April (*n* = 3), May (*n* = 1) and July (*n* = 1), and the remaining positive was from Walnut, CA in June. The only *Onchocerca*-positive *S. vittatum* (*s.l*.) was collected in San Dimas, California in April.

From the 2016 samples, all 43 individual heads of *S. tescorum* and 67 pools of heads of *S. vittatum* (*s.l*.) were negative for *Onchocerca* DNA. Screening of heads was performed because they are likely to harbor third-stage larvae (L3) of *Onchocerca*, following recommendations for molecular xenodiagnosis/entomological surveillance of *O. volvulus* in blackflies in sub-Saharan Africa or Latin American foci [30].

A single pool of *S. vittatum* (*s.l*.) bodies collected in April from La Verne was confirmed positive for the uncharacterized *Onchocerca*. All bodies of *S. tescorum* were negative for *Onchocerca* DNA. The locations where *Onchocerca*-positive blackflies were found can be seen in Fig. 1.

### Blood-meal analysis

A subset 36 blackflies from the 2015 collections was screened for the host blood meal using PCR targeting the *16S* rRNA of vertebrates, with one positive for Cervidae DNA. A single *S. tescorum*, which was positive for *Onchocerca* DNA, resulted in a 195 bp fragment sequenced, is 99% identical to sequences of mule deer, white-tailed deer, mule deer and red brocket deer *Mazama americana* (Erxleben), with only a single nucleotide deletion. No PCR amplification was obtained from other 35 blackflies. Based on the sequence identity comparison using the BLAST tool, we could not unequivocally identify the host species. However, only the mule deer naturally occurs in the Los Angeles County, and southern California, USA.

### Phylogenetic analysis

**Fig. 2:**
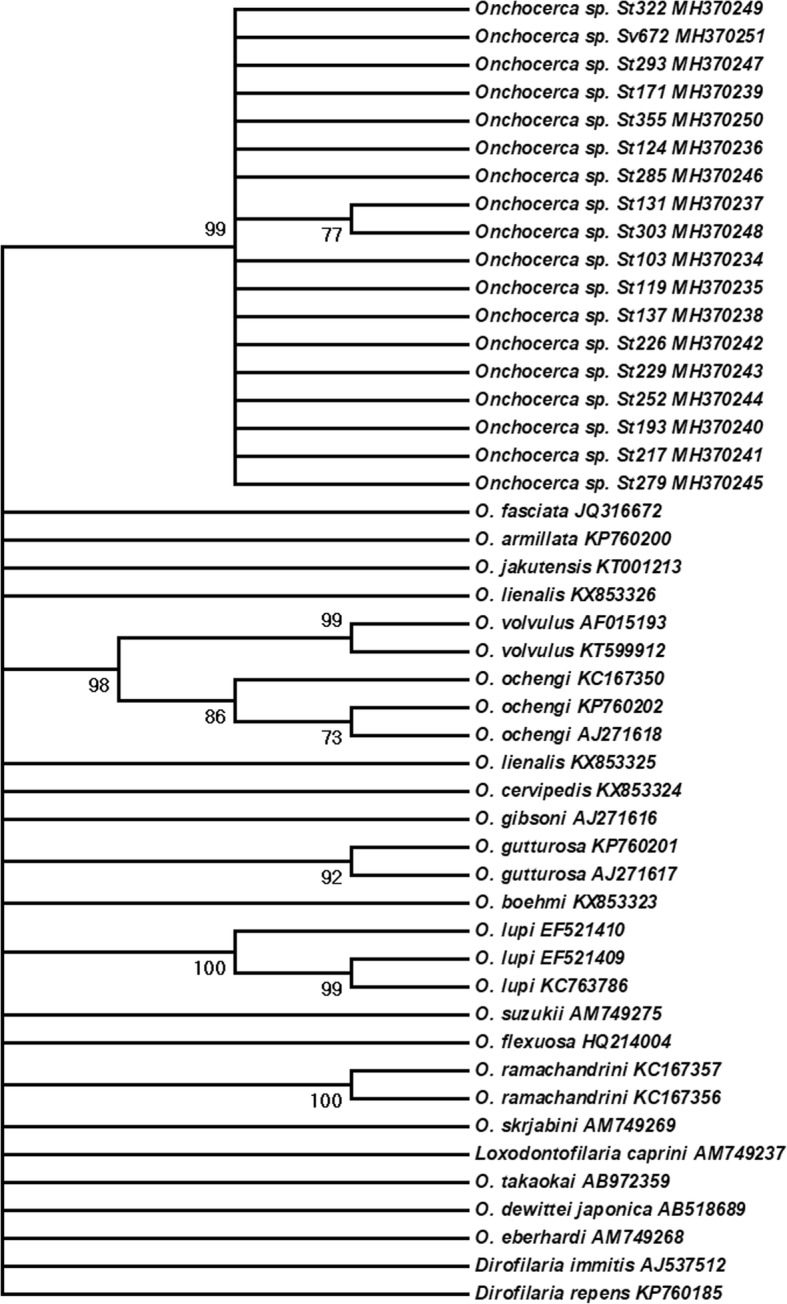
Maximum Parsimony tree depicting phylogenetic relationships among species of *Onchocerca* inferred from cytochrome *c* oxidase subunit 1 (*cox*1) mitochondrial gene. Branches with less than 70% bootstrap support were collapsed. Bootstrap support shown besides branches are based on 2000 replicates. All sequences labeled as *Onchocerca* sp. were obtained from blackflies from the Los Angeles County, southern California, and have been accessioned at GenBank (MH370234-MH370251). The codes *Sv* and *St* refer to *Simulium vittatum* (*s.l*.) and S. *tescorum*, respectively, from which each DNA sequence was obtained

All generated *cox*1 sequences were deposited in the GenBank database (MH370234-MH370251). The phylogenetic analysis, based on *cox*1 sequences, showed that all isolates formed a well-supported (99 bootstrap support), reciprocally monophyletic clade (Fig. 2), and belong to an uncharacterized species of *Onchocerca*. Pairwise distances among the *cox*1 sequences generated for the cryptic species varied from 0 to 0.012 (average 0.004), and there were only 6 variable sites.

Since there was no information on the *cox*1 sequences of the putative cryptic *Onchocerca* species in white-tailed deer from upstate New York, we also performed phylogenetic reconstruction based on the *nad*5 region. Pairwise-distance among the *nad*5 among LA isolates ranged between 0–0.025 (average 0.005), with 10 variable sites. Pairwise distances among the LA and NY isolates ranged between 0.011–0.016 (average 0.012), further supporting that these are conspecific.

**Fig. 3:**
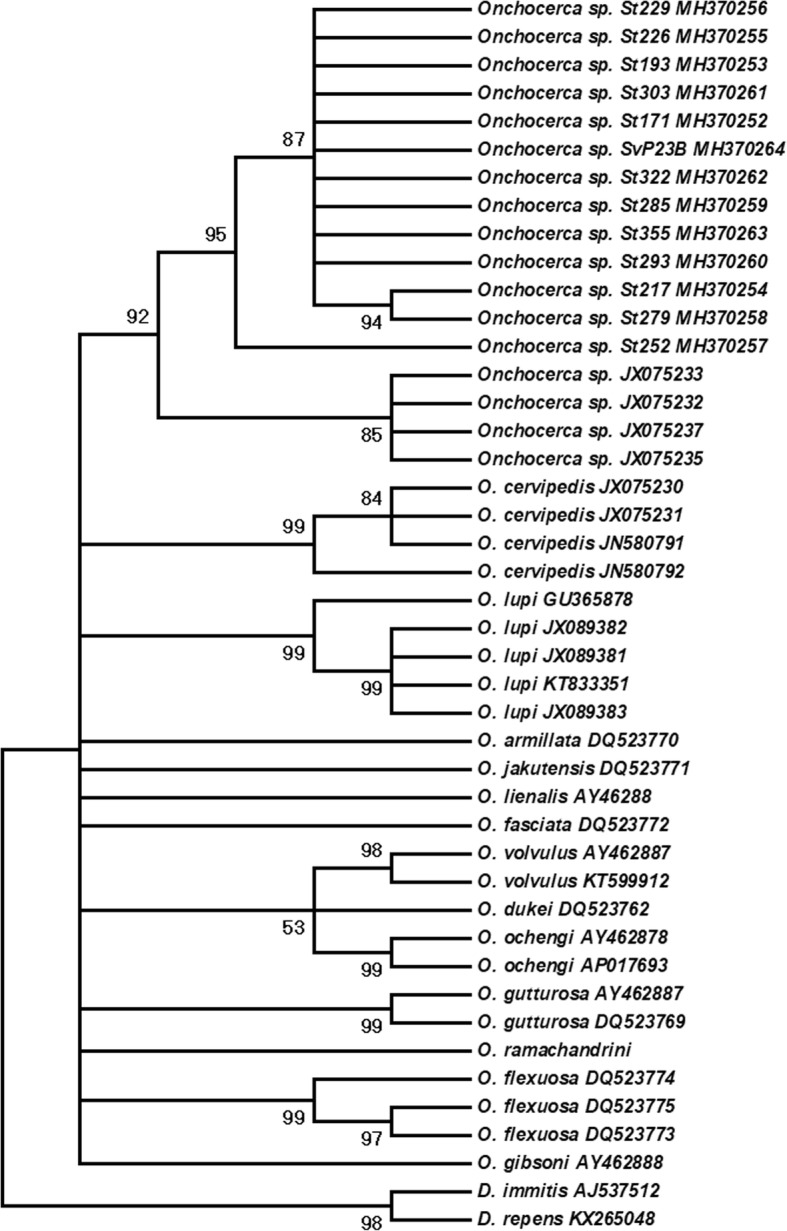
Maximum Parsimony tree depicting phylogenetic relationships among species of *Onchocerca* inferred from the NADH dehydrogenase subunit 5 (*nad*5) mitochondrial gene. Branches with less than 70% bootstrap support were collapsed. Bootstrap support shown besides branches are based on 2000 replicates. All sequences labeled as *Onchocerca* sp. followed by the codes *Sv* or *St* were obtained from blackflies from the Los Angeles County, southern California, and have been accessioned at GenBank (MH370252-MH370264). Sv and St refer to *Simulium vittatum* (*s.l*.), and S. *tescorum*, respectively, from which each DNA sequence was obtained. The code SvP23B refers to a pool of *S. vittatum* (*s.l*.) bodies. Sequences labeled as *Onchocerca* sp. followed by GenBank accession numbers JX075232, JX075233, JX075235 and JX075237 originated from upstate New York, and were made available through McFrederick et al. [7]

Similarly, all generated *nad*5 sequences were deposited in the GenBank database (MH370252-MH370264). Phylogenetic analysis based on *nad*5 sequences showed that all isolates formed a well-supported clade (95 bootstrap support). The sister group were the NY isolates, which also formed a well-supported clade (92 bootstrap support) (Fig. 3).

## Discussion

We have found DNA of a cryptic species of *Onchocerca* in two species of blackflies, *S. tescorum* and *S. vittatum* (*s.l*.) (*Simulium tribulatum* Lugger), from LAC, southern California, USA. Phylogenetic analyses demonstrated that these samples and populations appear conspecific with a recently discovered and undescribed species of *Onchocerca* infecting WTD in the northeastern USA [7]. Combined, these isolates from LAC and those from New York formed a well-supported monophyletic clade (92 bootstrap support) and demonstrated minimal divergence in *nad*5 sequences.

To date in North America only one species, *O. cervipedis*, has been formally described and has been reported to infect five species of cervids and the pronghorn [8, 11, 12]. Our work provides further evidence for the existence of a cryptic, undescribed species of *Onchocerca* associated with wild ungulates in North America [7]. *Onchocerca cervipedis* should now be recognized as a species complex; and hence, all previous host and geographical reports should be cautiously interpreted. The broad host range for *O. cervipedis* (*s.l*.) is atypical among species of *Onchocerca*, with only a few species known to infect multiple hosts [1]. The *O. cervipedis* species complex encompasses an extensive geographical distribution, ranging from tropical forests in Costa Rica, Central America [10] to subarctic environs of northern North America [11]. The original description of *O. cervipedis* by Wehr & Dikmans [8] was based on specimens collected from the subcutaneous tissues of the hindquarters, ankles and foot of two hosts and localities: white-tailed deer from Montana, USA, and Columbia black-tailed deer (*O. h. columbianus*) from British Columbia, Canada. There is a need to reassess the type-material of *O. cervipedis*, and to further characterize specimens collected from the two hosts, and ideally from the original localities using integrated classical and molecular tools. At this stage, it cannot be determined if either *O. cervipedis sensu* Verocai et al., 2012 [11] or the cryptic species isolated in the present study and in the skin of WTD in New York belong to *O. cervipedis* as originally described. The potential for zoonotic infections among species of *Onchocerca* establishes the need to characterize the recently recognized cryptic species, to explore for broader currently hidden diversity and to define the host and geographical limits within this assemblage.

In parallel, the recent discovery of a cryptic species of *Onchocerca* infecting white-tailed deer from upstate New York [7] was based on molecular evidence only. In contrast to our study, these conspecific parasites were revealed through sequencing of microfilariae in skin samples from deer. The effectiveness of surveillance based on screening of samples derived from definitive hosts or from arthropod vectors is established and provides the foundation to explore transmission dynamics from landscape to broader geographical scales. Although clearly established as a previously unrecognized species based on larval parasites, adult specimens in cervid definitive hosts have yet to be collected and described; formal description including all life history stages remains pending.

### Vector screening for cryptic diversity

Our results demonstrate that sampling and molecular screening of putative arthropod vectors is an effective path for recognizing cryptic vector-borne parasites. These methods provide for geographically widespread and site intensive sampling without requiring either invasive host sampling (i.e. in the case of *Onchocerca*, skin biopsy) or labor-intensive *post-mortem* examinations (i.e. fecal examinations for gastrointestinal and pulmonary parasites) [31, 32] Nevertheless, relevant geographical records, and putative information on host and vector associations may be acquired.

The overall prevalence of *Onchocerca* DNA in both blackfly species was low, but similar to the prevalence for *O. lupi* found in *S. tribulatum* from the same area [20]. Our findings suggest that *S. tescorum* and *S. vittatum* (*s.l*.) are putative vectors for this cryptic *Onchocerca*. Host-seeking blackflies are either autogenous seeking a blood meal after their first oviposition or have fed a few days ago (e.g. 72 h for *S. damnosum* (*s.l*.) [33]). The vast majority of collected flies were visually identified as not blood-fed, suggesting that the detected DNA could belong to larval stages undergoing development within the vector (L1 to L3), and not freshly ingested microfilariae. In addition, we failed to find *Onchocerca* DNA in the heads of both *Simulium* species from the 2016 collections, which could indicate the presence of infective L3 in the mouthparts of the fly. These negative results could confirm the competence of these blackfly species in transmission of the cryptic species of *Onchocerca*; hence these are considered putative vectors. However, the 2016 collections yielded much lower numbers of flies comparing to the 2015 collections, and overall low prevalence of *Onchocerca*.

Only a few blackfly species have been documented as putative vectors of *O. cervipedis* (*s.l.*) in North America; however, these few studies lack molecular confirmation of species identity of the nematode. *Prosimulium impostor* Peterson is the putative vector of a deer-associated *Onchocerca* in California [34], and *Simulium decorum* Walker and *Simulium venustum* Say are putative vectors for *O. cervipedis* among moose from northeastern Alberta, Canada [35]. The two putative vectors found in the present study differ significantly in their geographical distribution. Species within the *Simulium vittatum* complex, in particular *S. tribulatum*, are widely distributed across North America including areas of the northeastern USA where the cryptic *Onchocerca* has been reported [7, 22]. In contrast, the range of *S. tescorum* seems restricted to southern California and New Mexico [22], and therefore it may only play a role in transmission of the cryptic *Onchocerca* in the studied area. Nevertheless, since we now know that the North American *Onchocerca* diversity is higher than previously thought, the vector associations previously assumed for *O. cervipedis* should be reassessed across the continent using molecular methods. Concurrently, specimens of putative arthropod vectors should be routinely archived in museum collections as appropriate (e.g. when methods used are not destructive) as a basis to confirm identification and as baselines to allow assessment of ongoing ecological changes and shifts in distribution over time.

The presence of DNA of this cryptic *Onchocerca* species in blackflies also provides relevant information on the ecology of these blackflies, suggesting that both feed on cervids, with the mule deer being the only cervid endemic to LAC. *Simulium tescorum* is mammalophic and has been reported biting and potentially feeding on bighorn sheep (*Ovis canadensis* Shaw) and humans [36, 37]. The species of the *S. vittatum* complex in southern California is *S*. *tribulatum*, also a mammalophilic species, has been reported to feed on horses and cattle [22], and also to feed in ears of bighorn sheep [36]. This species is also known to feed on humans in the LAC (K. Nelson, personal communication). Our only successful blood-meal analysis showed DNA of *Odocoileus*, likely mule deer. Most studies on wild caught blackflies using CO_2_-baited traps are able to recover only a small percentage of blood-fed flies, and can be as low as 1 to 3–1000 flies [38, 39]. Indeed, our trapping strategy did not target blood-fed blackflies, which are in general less active than host-seeking flies, and less likely to be attracted to CO_2_ traps. The finding of blood-fed females may suggest an interrupted blood meal or that the fly was caught by chance (i.e. not necessarily attracted by the CO_2_). Also, the time after a blood meal may directly influence the sensitivity of a molecular assay. Blood meals ingested by *Simulium damnsosum* (*s.l*.) blackflies may not be detectable after two days due to complete digestion of blood and DNA degradation [33].

The only confirmed reports of another species of *Onchocerca*, based on molecular sequence data, in the southwestern USA, are for the zoonotic *O. lupi* [20, 40–44]. In the LAC, *O. lupi* was found in clinical canine cases of ocular onchocercosis and in *S. tribulatum*, which was then considered a putative vector for this species. Besides *O. lupi*, there have been reports of other species of *Onchocerca* associated with wild cervids (*Onchocerca jakutensis* (Gubanov, 1964)) and suids (*Onchocerca dewittei japonica* Uni, Bain & Takaoka, 2001) in humans, and therefore, other blackfly-transmitted *Onchocerca* species may also pose a potential zoonotic threat [45–47]. Climate envelopes may determine the geographical distributions of potential blackfly and related arthropod vectors, and accelerating climate warming and habitat change have considerable potential to modify the ranges of vector-borne host-parasite assemblages [48, 49]. Habitat disruption drives changing opportunities for transmission and may result in altered patterns of exposure and infection for species of *Onchocerca* among otherwise naïve and potential hosts such as other ungulates and humans. Opportunity and broad capacities for parasites to utilize host resources as defined by ecological fitting are anticipated to lead to broadened infection and emergence of disease in these and other host-parasite systems [50, 51]. Such considerations provide the rationale for continued efforts for the discovery of pathogen diversity, exploration of phylogeny and history, surveillance, monitoring and development of actionable information to limit the impact of emerging infectious disease in this arena of accelerating perturbation [52].

### Phylogenetic considerations, and evidence for a complex historical biogeography of *Onchocerca* in North America

Our intention was not to explore phylogenetic relationships in the broader context for the genus *Onchocerca* but to unequivocally identify this taxon in comparison with the two previously characterized isolates in North American cervids based on molecular data. Collectively, these constitute the limits so far recognized within the species complex for *O. cervipedis* [1, 7, 11]. In this limited comparative context, phylogenetic reconstruction based on *cox*1 and *nad*5 suggested that the cryptic *Onchocerca* species found in this study does not belong to the same taxon as *O. cervipedis* (*s.l.*) [11]. Further, in a recent phylogenetic study based on three mitochondrial genes (*nad*5, *12S*, and *16S*) [7], the cryptic species of *Onchocerca* was shown to be the putative sister taxon to *Onchocerca lienalis* (Stiles, 1892), a parasite of domestic cattle. This relationship could not be confirmed in the current analyses due to basal polytomies in both gene trees. The most comprehensive phylogenetic framework for the genus included *O. lienalis* among other cattle-associated species and in the same clade as *Onchocerca volvulus* of humans, and the zoonotic *O. lupi* [1]. Existing evidence supports the hypothesis that the two North American species of *Onchocerca*; i.e. *O. cervipedis sensu* Verocai et al., 2012 [11] and the cryptic species [7] represents the outcomes of independent expansion events with ungulate hosts across the Bering Landbridge from Eurasia in the Pliocene or Pleistocene [53]. Subsequent host associations may reflect a history of colonization and speciation. This pattern leading to assembly of a faunal mosaic is similar to that recognized among other mammalian- parasitic nematode assemblages in North America [53, 54], including *Varestrongylus* lungworms associated with cervids, and *Uncinaria* hookworms associated with bears, among others [55–61].

Based on geographical and putative host associations of the cryptic species of *Onchocerca*, we are able to make inferences on its historical biogeography. The existing records from two distant corners of the USA, southern California and upstate New York, support recognition of a widespread geographical distribution. Such reports also suggest that this species likely infects at least two species of *Odocoileus* including in areas in which these species do not co-occur. For example, the two subspecies of mule deer, *O. h. californicus* and *O. h. fuliginatus*, are endemic to southern California, and overall the species ranges across much of western North America, but is absent in the northeast [21]. In contrast, WTD is broadly distributed across eastern portions of western North America, excluding most of California, and extends southwards into northern South America [62].

## Conclusions

An uncharacterized *Onchocerca* species associated with cervid hosts was found in blackflies from southern California, USA. This species was previously found in WTD from upstate New York, and hence it supports a broad geographical distribution across North America. This considerable range, and knowledge of the geographical ranges of species of *Odocoileus* suggests that this cryptic *Onchocerca* may infect both white-tailed and mule deer. *Simulium tescorum* and *S. vittatum* (*s.l*.) (presumably *S. tribulatum*) of blackflies are putative vectors. The biodiversity, definitive host and vector associations of *Onchocerca* species in North America must be reassessed by means of a comprehensive, continental-scale study based on integrated classical and molecular approaches.
